# Exploring the Clinical Usefulness of Undergraduate Medical Research: A Mixed-Methods Study

**DOI:** 10.1007/s40670-024-02035-7

**Published:** 2024-04-20

**Authors:** Emma Burke, Colm Savage, John Begley, Stephanie Sioufi, Simon Smith, Slavi Stoyanov, Colm O’Tuathaigh

**Affiliations:** 1 School of Medicine, University College Cork, Cork, Ireland; 2 Medical Education Unit, School of Medicine, University College Cork, Cork, Ireland; 3 Open University of the Netherlands, 177, Valkenburgerweg 6401 DL Heerlen, The Netherlands

**Keywords:** Research-based learning, Clinical usefulness, Medical students

## Abstract

A significant amount of published clinical research has no measurable impact on health and disease outcomes, and research in undergraduate medical education is viewed as especially susceptible. The aims of this mixed methods study were to (a) to use group concept mapping (GCM) to explore key features identified by hospital physicians, medical educators, and medical students as central to clinical usefulness in an undergraduate medical research context, and (b) review a sample of undergraduate medical research projects based on usefulness criteria described by Ioannidis (2016). In the GCM procedure, 54 respondents (39 students, 15 physicians) from an Irish medical school participated across each of three phases: brainstorming, sorting, and rating. Data was analysed using multidimensional scaling and hierarchical clustering. A retrospective analysis of 252 student projects was also completed using a rubric based on Ioannidis’s (2016) six domains of “clinical usefulness”: problem base, context placement and information gain, pragmatism, patient-centredness, feasibility, and transparency. Projects were scored for each domain by three assessors. Results were analysed and presented using descriptive analysis.GCM analysis revealed the following “clinically useful” research characteristics: optimal design and methodology, practicality, research skills development, translational impact, patient-centredness, and asking a clinical question. Following a rubric-based analysis of projects, the highest scoring categories (mean rating; range of 1–4) were feasibility (3.57), transparency (3.32), and problem base (3.05). The lowest scoring areas were context placement and information gain (2.73), pragmatism (2.68), and patient-centredness (212). We identified considerable conceptual overlap between stakeholder consensus views on “clinical usefulness” as applied to undergraduate research and Ioannidis’s criteria. Patient-centredness was identified as a domain requiring greater emphasis during the design of undergraduate medical research.

**Supplementary Information:**

The online version contains supplementary material available at 10.1007/s40670-024-02035-7.

## Introduction

The development of research skills and attributes are vital elements of the undergraduate medical curriculum [1, 2]. Research in undergraduate medical education encourages the development of important transferable skills that are relevant for those who wish to pursue a career in medical research or a clinical practice orientated career. These core skills include the critical appraisal of evidence-based practice and new data, the understanding of how evidence-based practice is acquired, knowledge of the ethics and limits of published research, ability to work in a professional team, and the ability to communicate effectively with both patients and colleagues [2, 3]. The encouragement of active participation of students in research activities promotes their understanding of research methods and the benefits that research can provide them as either researchers and/or clinicians [1].

For clinical research to be valuable, it must provide findings that are not only accurate, but useful in the context in which they are presented [4]. Relating to the first criterion, it has been suggested that a significant amount of research currently published does not meet the criteria for being “true”—that most of it is false and/or exaggerated in its findings [4]. An estimation of 85% of resources allocated to the funding of clinical research has been described as “wasted” [4, 5]. Some argue that clinical research can be judged based on *practical impact*, and that a difference to health and disease outcomes should be a realistic prospect of proposed research [5]. Many in the scientific community have expressed broader concerns with regard to “evidence-based” clinical research and the way the research itself is being conducted, beyond the issue of wasted funds [6–8].

This criticism around research waste has been especially aimed at medical students. Some authors have consequently questioned the benefits to the student and medical schools of undergraduate research participation [9]. These concerns partly hinge on pragmatic concerns including costs, supervisor availability, and how such opportunities are provided within what is often a crowded curriculum [1]. Motivation to complete research at an undergraduate level is in many cases to remain competitive for job applications as opposed to academic interest. Often quantity is favoured over quality, with a higher number of publications seen as more desirable than one high-quality publication [9].

Ioannidis has described seven criteria that should be met for a study to be deemed clinically “useful” [10]. The first is a strong “problem base”—whether the research piece is addressing an issue of significant importance. The next category is “context placement and information gain”, where the production of relevant and impactful results depends on awareness and appraisal of previous research on the topic, and should be designed to provide sufficiently large amounts of evidence to support conclusions around relationship with clinical practice. Another category is “pragmatism”, where the study design should ensure that findings can be applicable in real-life, non-idealised circumstances. Valuable clinical research should be “patient-centred” where the benefit of the study should be solely to the patient’s health. The next category is “value for money,”, which may be difficult to capture, and useful clinical research should also be “feasible” and “transparent” with the data. To date, no studies have explored whether this conceptualisation of clinically useful research aligns with the perspectives of stakeholders involved in research in undergraduate medical education.

In their summative year, students at University College Cork medical school are required to submit a dissertation based on a longitudinal research project. Project supervisors, students, healthcare professionals, patients, and hospital administration staff all partake in the process of this dissertation project, inputting time and resources in ensuring the quality of these projects. The expected learning outcomes include the ability to apply scientific process and methodology to collect and analyse data that addresses a research question relevant to medicine [11, 12].

Employing a mixed-methods approach, the aims of this study were twofold. First, we used group concept mapping (GCM), a consensus-driven approach, to explore those key features deemed by hospital physicians, medical educators, and medical students to be central to the notion of “clinical usefulness” in an undergraduate medical research context. We sought to compare these features with Ioannidis’s criteria, in the expectation that the GCM analysis will identify commonalities as well as additional features specific to the undergraduate context. Secondly, we applied a rubric based on Ioannidis’s features of “clinically useful” research to a sample of over 250 medical student projects, to identify the applicability of these criteria to these projects, and to identify which of the seven categories were fulfilled more than others.

## Methods

### Study Design

#### GCM

GCM is a structured, mixed-methods approach using both qualitative and quantitative measures to explore consensus of various stakeholders around current understanding of a topic [13], in this instance what constitutes useful undergraduate medical student research. We employed simple random sampling of medical students (all from the final 2 years of the 5-year undergraduate degree programme or 4-year graduate-entry programme) attending University College Cork (UCC), and purposive and referral sampling of physicians and medical educators from the network of hospitals affiliated with the medical school. The primary outcome measure in this analysis was the list of topics derived from the GCM which respondents would include when asked to identify features associated with “clinically useful” undergraduate research. The secondary outcome measures were the ratings in terms of the criticality (i.e. importance) of each statement or topic to the concept of “clinically useful” research.

#### Retrospective Analysis of Research Reports

A grading rubric was developed by the study authors based on six domains described by Ioannidis and colleagues as crucial to “clinically useful research” (see Supplementary Material). The domains addressed were the following: problem base, context placement and information gain, pragmatism, patient-centredness, feasibility, transparency, and value for money. Projects obtained a score of 1 if they partially satisfied the question the criteria posed and a score of 4 if they clearly addressed all aspects of that criteria. Value for money was ultimately removed as a category due to difficulty quantifying the cost of each project. Additionally, the medical schools expect that most of the projects completed by students should be cost neutral. Pragmatism was divided into two separate categories, based on whether an observational or interventional design was used. The rubric was applied to a random sample of 252 clinical research projects as detailed in individual 5000-word reports formatted based on the standard “IMRaD” structure. All projects were completed between 2015 and 2017.

## Procedure

### GCM

This methodology involves three stages (brainstorming, sorting, rating), all of which were completed by participants using a web-based tool for data collection and analysis (Groupwisdom; https://groupwisdom.com). During “brainstorming”, participants were invited to answer a questionnaire with the prompt: “For an undergraduate medical student research project to be ‘clinically useful’, it should include the following characteristics”. The “sorting” stage consisted of sorting ideas into themes, based on similarity in meaning and giving names to the groups. In the rating stage, each statement was given a rating from 1 to 5 based on its perceived criticality to the concept of “clinically useful” research. All participants also submitted demographic and professional background information.

## Retrospective Analysis

Project reports were independently scored using the rubric by three trained assessors. Interrater reliability was quantified based on the degree of agreement between two or more coders who made independent ratings for 100 of the selected reports.

## Data Analysis

Following participants’ brainstorming, sorting, and rating, the data was analysed using the Groupwisdom concept mapping process platform. The software applies statistical techniques of multi-dimensional scaling and hierarchical cluster analysis, which quantitively aggregates individual inputs from participants to identify objective patterns in the data [14]. Results are produced in a visual format, which is a substantial part of the analysis. Visualization aids in grasping emerging data structures, interrelationships, and their interpretation [13]. The analysis of the data and interpretation of the results was then performed by the research team. Analysis was carried out to assign each cluster a bridging value, where a more homogenous group has a lower bridging value (i.e. greater coherence). Another important measure is the stress value, which indicates the goodness of fit of the configuration. A lower stress value means the data collected is a better representation of the data you would expect to find in the actual population, and means the results are interpretable with a large degree of certainty. Group concept mapping projects are generally expected to have a stress value between 0.205 and 0.365 [15].

When the rubric data was collated, descriptive statistics were employed to analyse the data for any presenting patterns and for overall distribution. The frequency of each overall score and the frequency of each score of each variable were calculated. Cohen’s kappa (*κ*) was used estimate the level of intercoder reliability for a subset (*N* = 100) of the selected projects. Interpretation of Cohen’s kappa results was as follows: values ≤ 0, no agreement; 0.01–0.20, none to slight; 0.21–0.40, fair; 0.41–0.60, moderate; 0.61–0.80, substantial; 0.81–1.00, near perfect agreement.

Both study components were approved by the School of Medicine Sub-committee of the Social Research Ethics Committee of University College Cork.

## Results

### GCM Study

Of the 381 UCC medical students who were invited to the study, 53 students (27 male, 26 female) accepted the invitation and completed the socio-demographic factor questionnaire. Eight hospital-based physicians (non-consultant hospital doctor and consultant) and 14 medical educators (six of whom had a non-medical academic background) also agreed to participate.

Twenty-two of these participants, eight medical educators and 14 medical students, agreed to complete the sorting stage (stage 2), and 20 participants (seven medical educators, 13 medical students) completed the rating stage (stage 3). This sample size across both stages is sufficient to produce valid results, where the recommended sample size for both stages is at least 15 participants [16].

**Fig. 1:**
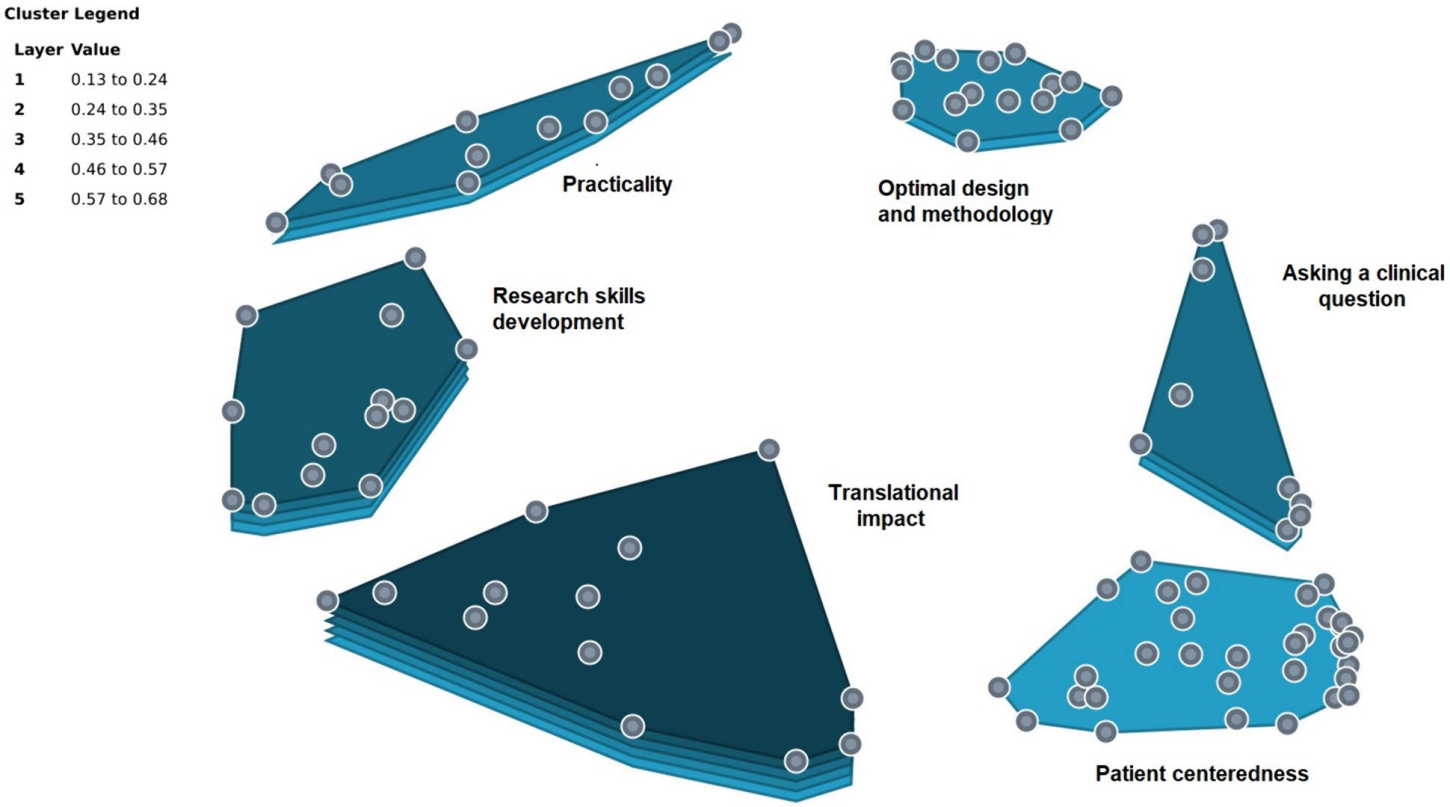
Six-cluster solution with the following domains: Optimal design and methodology; Practicality; Research skills development; Translational impact; Patient centredness; Asking a clinical question. Layers refer to mean bridging values

Six clusters representing characteristics associated with a clinically useful undergraduate medical student research are presented in Fig. 1 and summarized descriptively in Table 1. A full list of clusters and all associated statements is available in Supplementary Materials. This study has a stress value of 0.22, which is considered adequate internal validity in comparison with the stress values in a meta-analysis of 69 group concept mapping projects [16]. With respect to the GCM cluster map (Fig. 1), the closer the points are to one another, the more often that participants sorted these statements together and the farther they are from one another, the more distinct they are.

**Table 1:** Six clusters with names, mean bridging values, definitions and sample statements for each

	**Title and definition**	**Mean bridging value**	**Number of statements**	**Sample statement(s)**
1	**Cluster 1: Optimal design and methodology** e.g. Well-designed studies with clearly defined outcome measures	0.26	16	“A well-researched literature review”, “Have clear quantifiable outcomes”, “The question should be evidence based”
2	**Cluster 2: Practicality** e.g. That the project should be appropriate and achievable for an undergraduate student	0.37	13	“Project should be realistic to achieve in the timeframe”, “Achievable in the timeframe given and in terms of the resources and skills required”
3	**Cluster 3: Research skills development** e.g. Afford the student the opportunity to learn and develop research skills in clinical medicine	0.53	13	“Focus on the process and learning the appropriate skills for conducting high quality and clinically research the undergraduate project may not be clinically useful but the skills developed are”, “It should be a topic that is useful to the student for their chosen career interest”
4	**Cluster 4: Translational impact** e.g. Ability to translate findings to clinical practice	0.68	13	“Should add to the current body of literature”, “To be clinically useful, undergraduate medical student research should have a broad base and a wide reach in terms of impact via dissemination”, “The research needs to disseminated to the right target audience”
5	**Cluster 5: Patient centredness** e.g. Multiple angled stakeholder involvement, and with a view to improving patient-centred care	0.13	32	“It needs to be patient focused”, “Any topic that looks at ways to improve the patient experience whether it be through social, psychological, or medical interventions”, “Pragmatic and add to the knowledge base clinical care”
6	**Cluster 6: Asking a clinical question** e.g. Selecting a research question based on a knowledge gap identified by literature review	0.44	9	“Address a real world problem/issue”, “It has to address gaps in the literature”, “..a very clear research question so that information that is collected can.. guide or suggest small changes in practice that will benefit patients, healthcare professionals or both”

Greatest coherence (lowest bridging values) was observed for the “patient centredness” and “optimal design and methodology” clusters, while the lowest coherence (i.e. highest bridging value) was observed for the “translational impact” cluster. When statements were rated by participants based on perceived criticality (1, not critical; 5, very critical) “optimal design and methodology” strategy received the highest mean rating (4.23), followed by “pragmatism” (3.93), “asking a clinical question” (3.58), and “research skills development” (3.45). The two lowest ranked clusters were “patient centredness” (3.38) and “translational impact” (3.03).

## Rubric-Based Assessment of Clinical Usefulness of Undergraduate Medical Projects

Descriptive statistics for each of the six criteria measured in the rubric are presented for 252 research projects in Table 2. With regard to clinical area, 54% (*N* = 136) were medical (including emergency medicine) and 22% (*N* = 56) were surgical (including anaesthetics and pain medicine) projects, with all remaining projects (24%, *N* = 60) distributed across paediatrics, obstetrics and gynaecology, and psychiatry. Ninety-six per cent of projects had an observational design (*n* = 242), while 4% of projects were intervention-based (*N* = 10). Most projects were quantitative at 81% (*N* = 204), 9% (*N* = 23) were qualitative, and 10% (*N* = 25) were mixed-methods studies. Acceptable interrater reliability was reported, as indicated by fair to moderate *κ* values reported for all six domains (Table 2).

**Table 2:** Descriptive statistical summary of scores awarded to student projects (*N* = 253) across the six Ioannidis domains, as well as Cohen’s kappa (*κ*) values for each domain

	Mean	SD	Median	κ
Problem base	3.05	0.89	3.0	0.39
Context placement and information gain	2.73	0.94	3.0	0.32
Pragmatism	2.68	0.88	3.0	0.44
Patient centredness	2.12	0.55	2.0	0.57
Feasibility	3.57	0.56	4.0	0.54
Transparency	3.32	0.72	3.0	0.42

Less than 1% (*N* = 1) or 5% (*N* = 11) failed to satisfy the criteria at least partially (i.e. score a “1”) for the “feasibility” and “transparency” domains, respectively. Similarly, a little over 5% of projects (6%, *N* = 15) received a score of 1 for the “problem base” score, indicating that most papers had at least addressed a non-trivial medical research question. The majority (75%, *N* = 189) of papers had “good” (3) or “very good” (4); a score of 3 was given when the research addressed specific issues and highlighted the existing knowledge gaps in the literature. However, only 35.7% (*N* = 90) stated the consequences if the problem is not resolved, or the benefit(s) if resolved. A more equal distribution was observed for the “context placement and information gain” and “pragmatism” domains, where 57.5% (*N* = 145) and 57.9% (*N* = 146) received “good” or “very good” scores respectively. The lowest scoring domain was “patient centredness”, with 78.9% (*N* = 199) of projects receiving a score of “1” or “2”, indicating inadequate performance in this area. As a whole, 0.8% (*N* = 2) scored “good” or “very good” for only one of the six criteria, with 16.3% (*N* = 41) receiving same for only two criteria, 15.1% (*N* = 38) for three criteria, 24.6% (*N* = 62) for four, 35.3% (*N* = 89) for five, and 7.9% (*N* = 20) for all six criteria.

## Discussion

This study describes the application of the concept mapping approach to develop a consensus on stakeholders’ understanding of what constitutes clinically useful undergraduate medical research. Comparison of the clusters identified in our GCM analysis with six qualities of useful clinical research developed by Ioannidis [10] reveals considerable overlap (see Table 3). The “optimal design and methodology” domain ranked strongest in both criticality and cluster coherence, and maps on to Ioannidis’s “context placement and information gain”, “feasibility”, and “transparency” features. Similarly, two of the GCM clusters “patient centredness” and “practicality” are closely thematically aligned with the identically named “patient centredness” and “feasibility” domains respectively. Both the GCM-identified clusters “asking a clinical question” and “optimal design and methodology” are aligned with Ioannidis’s “problem base” criterion. Similarly, both “pragmatism” (Ioannidis) and “translational impact” (GCM) are focused on ecological validity and relevance of the design to clinical practice, while “translational impact” also emphasises the importance of a dissemination strategy which is practice-changing in its ambition. The “research skills development” cluster does not have a parallel with any of Ioannidis’s features, as it relates specifically to the benefits to the student of engaging in clinical research activity. It is argued that provision of opportunities to medical students for hands-on experience in research provides numerous benefits ranging from a personal sense of achievement and the ability of critical appraisal of research, communication skills in understanding, and dissemination of information, all of which have a direct impact on their responsibilities as a future physician [17]. Direct experience in the elaboration of the methodology of a research study both allow for future research to be conducted and also shed insight on the appraisal of clinical research methodology, which is the basis of evidence-based medicine. Others have argued that though student research can be useful if conducted under adequate conditions, it is not necessary [18]. In the current study, where “research skills development” scored highly with respect to criticality (average rating of 3.45/5), statements revealed the benefit to the student of developing familiarity with handling authentic clinical data and interacting with patients in a research context.

**Table 3:** Representation of relationship between GCM clusters identified in present study and the six Ioannidis domains

**Ioannidis (2016) domains**	**GCM-identified domains**
Problem base	Asking a clinical question
Context placement and information gain	Optimal design and methodology
Pragmatism	Translational impact
Patient centredness	Patient centredness
Feasibility	Optimal design and methodology Practicality
Transparency	Optimal design and methodology

In the GCM analysis, a lower bridging value means that each statement within the theme is more closely associated with the other statements in the same theme, i.e. a more homogeneous group and higher coherence [16]. The clusters that were least coherent were “translational impact” (bridging value 0.68) and “research skills development” (bridging value 0.53). Respondents differed greatly on their definition and measurement of impact as it relates to undergraduate medical research; a divergence of views was also observed for what skills should be fostered (or emphasised) during research project participation. The most coherent clusters were “patient centredness” (bridging value 0.13) and “optimal design and methodology” (bridging value 0.26). The criticality rating based on the six-cluster solution demonstrates the themes perceived as most important to characterizing “clinically useful” undergraduate medical projects. The areas with the highest score were “optimal design and methodology” (average rating of 4.23/5) followed by “pragmatism” (3.93). This pattern likely represents acknowledgement of the limitations, in terms of scope and resources available, and the ongoing trade-offs between pragmatic concerns and research impact. For example, the parameters of the study must be compatible with a medical student’s other academic and clinical training commitments, while still giving them the opportunity to learn new skills and interact with their project supervisor.

Useful clinical research should address patient priorities to benefit their health. Our rubric-based review of previously completed student projects showed that “Patient centredness” was the lowest ranked domain. This finding complements the low prioritization (i.e. low criticality rating) for this criterion by the various groups in our GCM analysis. Our retrospective analysis revealed that only a single paper included patients in the design of the study and contacted patients after the research was complete to capture patient satisfaction levels. Patient centredness has a positive transformative impact on research [19, 20], where their perspectives and lived experiences contribute to the usefulness of research and help focus the research question [21]. Involving patients in the research design often may shift the research outcomes and goals so that they align better with patient priorities. For example, traditional outcomes such as cure or longer survival may not be as relevant to the patient as disease burden or quality of life. If undergraduate research aims to improve care the outcomes of the treatments or procedures provided, then it must align itself with issues and interests relevant to patients themselves. Resource-efficient solutions might involve including patients in the question and/or design formulation steps to ensure that the study aligns with patient values and what patients perceive to be important outcomes. Following up with patients’ post-study to obtain their perspectives, opinions, and experiences with the study is another step in this direction. Another way for research to become more patient centred involves the use of Patient-Reported Outcomes Measures (PROMs). PROMs were originally used to obtain patient feedback in clinical trials but are now becoming more widespread among other forms of clinical research. PROMs consist of standardised questions that attempt to depict a patient’s quality of life and their perception of their health [22]. Incorporating PROMs will contribute to making undergraduate research more patient centred, contributing to the overall clinical usefulness of this activity.

Out of the six domains measured in the retrospective analysis of student projects, “feasibility” and “transparency” scored the highest. With respect to “feasibility”, these projects may have scored highly in this area as most projects were designed in conjunction with an experienced clinical project supervisor, and they would have ensured that the project was within the student’s capabilities. Due to the project completion schedule (i.e. requirement for completion within 24 months), the study design was heavily skewed towards cross-sectional studies or retrospective review(s) of patient notes or clinical databases. Conversely, due to the limited timeframe, as well as the absence of any dedicated school funding for individual projects, students would be less likely to embark on prospective cohort or intervention-based studies. Therefore, projects would be less likely to be discontinued due to lack of funds or loss to follow-up, increasing the “feasibility” score across the board. One of the limitations of this analysis was the limited diversity of study designs employed in the sample of student projects, where most were observational studies. A larger, more diverse sample would allow a more detailed comparison of the relationship between study design, specialty, etc., and scoring across the identified criteria. Future studies might also compare ratings of student projects using the present rubric between clinicians of varying levels of clinical experience and research activity. Transparency also scored highly, as many project papers included data required to validate results in the main body or appendices. This result differs from reports that this level of transparency is generally uncommon, occurring only in 1–20% of all clinical research [10]. While value for money was not a criterion that was scored in these projects, it would have scored quite highly, positively impacting project usefulness. These projects are usually inexpensive to carry out, usually designed in such a way that data can be accessed for free, and that patients are not paid for participation.

In conclusion, we demonstrate that research projects carried out by medical students contain many aspects of what is considered “useful” clinical research. Physicians in general, across the world, are expected to be involved in publishing research papers to some degree. An issue arises when such physicians are evaluated on the number of published papers rather than the quality of the work [23]. As one would expect, this leads to the mass production of papers that might not necessarily have a strong clinical impact [10, 23]. Student research is an important piece of the puzzle as it is often a steppingstone for young physicians considering careers in academic medicine. Students should be aware of areas of clinical usefulness when designing future research projects and how their projects address these criteria. It is recommended that undergraduate courses designed to enhance the research skills of undergraduate medical students, particularly those which include completion of research projects, should incorporate consideration of criteria for clinical usefulness during research project conceptualisation and development. Incorporation of priorities related to clinical usefulness at the project conceptualization and design stages will provide an opportunity for students to learn about the importance of high-quality clinical research activity, and an opportunity for supervisors and faculty to assess the extent to which projects under consideration are a valuable use of time and resources. Additionally, the results of the GCM analysis identified relevant features specific to undergraduate research (e.g. research skills development) which should be addressed in the development of novel tools to define and measure the clinical utility of undergraduate research projects. Our data identifies patient centredness, in particular, as an area that if taken into consideration to a greater degree by medical students, would contribute to the overall clinical usefulness of their research projects.

### Supplementary Information

Below is the link to the electronic supplementary material.

Supplementary file1 (DOCX 33 KB)

## Data Availability

Data are available on reasonable request.
