# Supplementary material for: Exploring the Clinical Usefulness of Undergraduate Medical Research: A Mixed-Methods Study
Source: Med Sci Educ. 2024 Apr 20;34(4):823–30. doi: 10.1007/s40670-024-02035-7 (PMC11296999; doi:10.1007/s40670-024-02035-7)
Supplement: Supplementary file 1 — Supplementary file1 (DOCX 33 KB) [file 40670_2024_2035_MOESM1_ESM.docx]

**SUPPLEMENTAL MATERIAL**

**Supplementary Data 1**

**Rubric**

| **Features** |
| --- |
| Problem base |
| Context placement and information gain |
| Pragmatism |
| Patient centredness |
| Feasibility |
| Transparency |

**1 - Problem Base**

| 1 | 2 | 3 | 4 |
| --- | --- | --- | --- |
| Research does not clearly address a specific issue, difficulty, contraindication or any existing knowledge gaps. | Research addresses a specific issue, difficulty, contraindication, or existing gap in knowledge. However, the problem is non specific (place, time and people) and/or has low prevalence/disease burden. | Research addresses a specific issue, difficulty, contraindication, or existing gap in knowledge. The problem is specific (place, time and people) and relevant.  There are no consequences if the problem is not resolved and/or no benefit if the problem is resolved. | Research clearly addresses a specific issue, difficulty, contraindication, or existing gap in knowledge. The problem is specific (place, time and people) and relevant. There are consequences if the problem is not resolved and/or there is benefit if the problem is resolved. |

| Addresses knowledge gap | Yes/No |
| --- | --- |
| Problem specific or high prevalence/disease burden | Yes/No |
| Consequences if problem not resolved or benefit if resolved | Yes/No |

**2 - Context placement and information gain**

| 1 | 2 | | 3 | | 4 | |
| --- | --- | --- | --- | --- | --- | --- |
| Prior evidence has not been sufficiently systematically assessed. (Literature review has not been completed) | | Prior evidence has been sufficiently systematically assessed to inform the need for new studies. However, research does not procure a clinically relevant information gain that adds to what we know. | | Prior evidence has been sufficiently systematically assessed to inform the need for new studies. Research procures a clinically relevant information gain that adds to what we know so information gain can be placed in context.  Study is designed to provide sufficiently large amounts of evidence to ensure patients, clinicians, and decision makers can be confident about the magnitude and specifics of benefits and harms. | | Prior evidence has been sufficiently systematically assessed to inform the need for new studies. Research procures a clinically relevant information gain that adds to what we know so information gain can be placed in context.    Study is designed to provide sufficiently large amounts of evidence to ensure patients, clinicians, and decision makers can be confident about the magnitude and specifics of benefits and harms.    Study is clinically useful regardless of their eventual results. |

| Literature review completed | Yes/No |
| --- | --- |
| Clinically relevant information gain | Yes/No |
| Ability to change practice | Yes/No |
| Clinically useful regardless of results | Yes/No |

**3.1 - Pragmatism (Intervention based study)**

| 1 | 2 | 3 | 4 |
| --- | --- | --- | --- |
| Not directly relevant to participants, communities, and healthcare practitioners.  Little effort is made to match the design of the trial to the decision-making needs of those in the usual setting in which the intervention will be implemented.  Idealised clinical trial circumstances  Well resourced, 'ideal' setting. Highly selected; poorly adherent participants and those with conditions which might dilute the effect are often excluded  Casual research hypotheses tested- causative explanation for an outcome | Directly relevant to participants, communities, and healthcare practitioners.  Little effort is made to match the design of the trial to the decision-making needs of those in the usual setting in which the intervention will be implemented.  Idealised clinical trial circumstances  Well resourced, 'ideal' setting  Highly selected; poorly adherent participants and those with conditions which might dilute the effect are often excluded.  Casual research hypotheses tested- causative explanation for an outcome | Directly relevant to participants, communities, and healthcare practitioners.  The trial is designed to meet the needs of those making decisions about treatment options in the setting in which the intervention will be implemented  Little or no selection beyond the clinical indication of interest, real life circumstances captured.  Casual research hypotheses tested- causative explanation for an outcome | Directly relevant to participants, communities, and healthcare practitioners.  The trial is designed to meet the needs of those making decisions about treatment options in the setting in which the intervention will be implemented  Little or no selection beyond the clinical indication of interest, real life circumstances captured.  Focus on correlation between treatments and outcomes in real world health system practice and is designed to help choose between care options. Effectiveness and effect of treatment in routine clinical practice evaluated |

| Directly relevant to participants, funders, communities or health care practitioners | Yes/No |
| --- | --- |
| Treatments affect real world practice | Yes/No |
| Real life circumstances captured | Yes/No |
| Helps choose between care pathways | Yes/No |

**3.2 - Pragmatism (Observational Study)**

| 1 | 2 | 3 | 4 |
| --- | --- | --- | --- |
| Not directly relevant to participants, communities, and healthcare practitioners.  Participants not representative of usual clinical practice.  Characteristics that influence validity have not been accounted for. (Information bias, selection bias and confounding bias)*  Research hypotheses tested- causative explanation for an outcome | Directly relevant to participants, communities, and healthcare practitioners.  Participants not representative of usual clinical practice.  Characteristics that influence validity of study have been accounted for.  (Information bias, selection bias and confounding bias)*  Research hypotheses tested- causative explanation for an outcome | Directly relevant to participants, communities, and healthcare practitioners.  Participants representative of usual clinical practice.  Characteristics that influence validity have been accounted for.  (Information bias, selection bias and confounding bias)*  Research hypotheses tested- causative explanation for an outcome | Directly relevant to participants, communities, and healthcare practitioners.  Participants representative of usual clinical practice.  Characteristics that influence validity have been accounted for.  (Information bias, selection bias and confounding bias)*  Study is designed to help choose between care pathways. |

***Characteristics That Influence Validity**

**Information bias-** Incorrectly ascertaining treatment or outcome

**Selection bias**- Pretreatment – subjects on different treatments have different risks

   Post treatment – lost to follow-up depends on outcome and treatment

**Confounding Bias -** Caused by pretreatment selection bias

   Demonstrated by differences in risk factors between treatment groups

  Possibly reduced by risk-adjustment

| Directly relevant to participants, communities and healthcare practitioners. | Yes/No |
| --- | --- |
| Participants representative of usual clinical practice. | Yes/No |
| Attempt made to account for bias | Yes/No |
| Helps choose between care pathways | Yes/No |

**4 - Patient Centeredness**

| 1 | 2 | 3 | 4 |
| --- | --- | --- | --- |
| Research does not benefit patients (preserve health and enhances wellness). | Research benefits patients (preserves health and enhances wellness). Patients not consulted with research design and not contacted with feedback/Survey result | Research benefits patients. Patients consulted with research design, research is aligned with patient priorities. Research questions address what is important to patients. | Research is done to benefit patients. Patients involved throughout research process. Patients consulted in research design process, research is in line with patient priorities. Patients contacted after research is complete patients receive feedback regarding results of survey and patient satisfaction levels captured. |

| Research benefits patients | Yes/No |
| --- | --- |
| Patients consulted | Yes/No |
| Patients contacted after research completion | Yes/No |

**5 - Feasibility**

| 1 | 2 | 3 | 4 |
| --- | --- | --- | --- |
| Research was discontinued, no data collection. | Research has not been completed, incomplete data collection | Data collection complete but insufficient data collected for any conclusions to be drawn, or statistically insignificant results | Research has been completed in full and full data set has been analysed with conclusions drawn from the data collected |

| Research completed | Yes/No |
| --- | --- |
| Incomplete data collection | Yes /No |
| Insufficient data collection for conclusion/ statistically insignificant | Yes/No |
| Full data set analysed with conclusions drawn/statistically significant result | Yes/No |

**6 - Transparency**

| 1 | 2 | 3 | 4 |
| --- | --- | --- | --- |
| Full raw study data unavailable | Full raw data set available, Full data set not analysed, | Full raw data set available, full data set analysed, selective reporting of study results | Full raw study data available to public, full data set analysed in research paper, all data results reported |

| Raw data set available | Yes/No |
| --- | --- |
| Selective reporting of results | Yes/No |
| Full data set reported and analysed | Yes/No |

**Supplementary Data 2**

# Statements and Cluster Report

| **CLUSTER SOLUTION** | **STATEMENTS** | | **BRIDGING VALUES** |
| --- | --- | --- | --- |
| **Optimal Design and Methodology** | | | 0.26 |
|  | 1 | Well-designed studies with clearly defined outcome measures | 0.25 |
|  | 19 | A well-researched literature review | 0.43 |
|  | 20 | A sufficient number of people in the study, a good design and a simple straightforward well formed research question | 0.24 |
|  | 22 | Thorough, with sound methodology. | 0.2 |
|  | 27 | The research should have sound methodology such that the findings are reliable and valid | 0.27 |
|  | 38 | It should be unbiased | 0.23 |
|  | 43 | The project should be well-designed to ensure reproducible results | 0.18 |
|  | 58 | It should be evidence based. | 0.26 |
|  | 65 | external validity. | 0.37 |
|  | 68 | Accurate and appropriate statistics | 0.24 |
|  | 80 | Be reproducible in a larger confirmatory study | 0.2 |
|  | 82 | Conducted in a robust manor, using analysis which allows inference | 0.22 |
|  | 84 | basic in nature, robust in terms of methodology to result in meaningful findings. | 0.24 |
|  | 85 | possess simple and identifiable outcomes. | 0.36 |
|  | 90 | Have clear quantifiable outcomes | 0.22 |
|  | 95 | Focused objectives | 0.28 |

| **Cluster solution** | **STATEMENTS** | | **BRIDGING** |
| --- | --- | --- | --- |
| **Practicality** | | | 0.37 |
|  | 11 | Have a supervisor able to identify research questions capable of being addressed within the timescale and resources available to the undergraduate | 0.41 |
|  | 14 | Should be at an appropriate level of complexity for the students - ie not too complex but equally not too basic. | 0.43 |
|  | 23 | Needs to be feasible to complete it in the timescale and with the resources available to the student | 0.36 |
|  | 33 | It should be cost neutral | 0.33 |
|  | 37 | It should be cost efficient. | 0.3 |
|  | 44 | Project should be realistic to achieve in the timeframe | 0.3 |
|  | 47 | good support and guidance from project supervisor at all stages of the project - protocol development, data collection, data analysis, write-up | 0.42 |
|  | 48 | Senior oversight into developing a suitable protocol, support with data collection and analysis, and guidance on data interpretation and manuscript write up. | 0.45 |
|  | 54 | Can be conducted in a reasonable amount of time and facilitates data collection at reasonable times e.g a project may be unreadable if data can only be collected during weekdays while we have ongoing placement | 0.3 |
|  | 60 | It should be clearly defined as a project suitable and realistic for a full time medical student. | 0.38 |
|  | 63 | Clearly defined scope, appropriate for an undergraduate student to undertake | 0.37 |
|  | 67 | Achievable in the timeframe given and in terms of the resources and skills required | 0.3 |
|  | 72 | Students have no idea what research is truly meaningful/novel. So you definitely need a good supervisor to make sure you are on track, not just in terms of completing tasks - because we're all adults and can do that - but to make sure you're relevant | 0.45 |

| **CLUSTER TITLE** | **STATEMENTS** | | **BRIDGING VALUE** |
| --- | --- | --- | --- |
| **Translational impact** | | |  |
|  | 5 | To be clinically useful, undergraduate medical student research should have a broad base and a wide reach in terms of impact via dissemination | 0.57 |
|  | 16 | Should add to the current body of literature | 0.82 |
|  | 21 | Presented / listened to / reviewed and possibly published . We all have a lot to learn from tstudents . If it is soundly done all research can then be clinically useful to one/ some/ many . | 0.73 |
|  | 29 | The research needs to disseminated to the right target audience | 0.94 |
|  | 30 | The research questions should be developed with a clinician supervisor | 0.72 |
|  | 34 | It should be relevant to clinical work students are part of. | 0.61 |
|  | 39 | It should be innovative | 0.66 |
|  | 49 | Be relevant to the students' professional interests. | 0.61 |
|  | 52 | It should focus on common/broad scenarios/medical conditions that can be appreciated by medical students. | 0.62 |
|  | 75 | Practical skills based components | 0.64 |
|  | 79 | Likelihood of publication | 0.83 |
|  | 86 | improve a students clinical skills. | 0.6 |
|  | 93 | Transferable skills Interaction between clinical and laboratory work | 0.54 |

| **Cluster solution** | **STATEMENTS** | | **BRIDGING** |
| --- | --- | --- | --- |
| **Research Skills Development** | | | 0.53 |
|  | 12 | It should be a topic that is useful to the student for their FYP, elective and chosen career interest, if known. | 0.57 |
|  | 15 | For the student's sake, it should be something that they themselves are interested in. | 0.53 |
|  | 17 | Strong supervisor who is themselves research active so that the project will be relevant and worthwhile. | 0.64 |
|  | 25 | Involve a senior clinician | 1 |
|  | 35 | It is a great benefit for both the student and supervisor to be fully invested in the project. | 0.5 |
|  | 45 | at an appropriate level for the students knowledge | 0.49 |
|  | 46 | Focus on the process and learning the appropriate skills for conducting high quality and clinically research the undergraduate project may not be clinically useful but the skills developed are important for future research projects | 0.41 |
|  | 50 | Should allow the student to learn how to carry out data analysis through a new method (e.g. meta-analysis) | 0.42 |
|  | 51 | Offer students the opportunity to expand different research skills such as interacting with patients as a researcher (e.g. filling out a survey, conducting an interview) | 0.4 |
|  | 59 | Involved Informative Hands on | 0.52 |
|  | 66 | Teach students about different type of study bias | 0.44 |
|  | 74 | Afford the student the opportunity to learn and develop research skills in clinical medicine | 0.44 |
|  | 76 | It should provide experience in how to conduct and write a research study in the everyday world. | 0.49 |

| **Cluster solution** | **STATEMENTS** | | **BRIDGING** |
| --- | --- | --- | --- |
| **Pragmatism** | | | 0.13 |
|  | 2 | Research which has a clear relationship with patient welfare | 0.05 |
|  | 3 | To complete research which will inform local clinical practice | 0 |
|  | 4 | To be clinically useful, perhaps teams of stakeholders should be brought together (management, clinical practice /teaching /research, patients) to help medical students develop research questions/outcomes that would impact on policy and practice | 0.11 |
|  | 6 | Clinical usefulness would include all stakeholders within a clinical service system so research on clinical management / healthcare services also relevant | 0.07 |
|  | 7 | To be clinically useful, research-based evaluations of clinical teaching and clinical research development are as important as clinical practice-based research outcomes for the benefit of patients or service users | 0.11 |
|  | 8 | Clinical usefulness can of course include clinical research development, clinical teaching or clinical practice-based perspectives - a wide lens | 0.23 |
|  | 9 | It should have outcome measures that indirectly or directly affect clinical practice | 0.01 |
|  | 10 | Address a clinically relevant question Be applicable to current practice Inform and ideally improve clinical practice - i.e. in the case of a quality improvement project Ideally be disseminated locally and nationally/internationally Be interesting | 0.07 |
|  | 13 | Ideally it should be of interest to clinicians in general - not some supervisor's niche topic that no one else is interested in. | 0.29 |
|  | 18 | Should be relevant to clinical practice | 0.03 |
|  | 26 | Involvement of stakeholders including patients - strengthens the likelihood that research will be 'useful'. | 0.05 |
|  | 28 | The findings should help at least one clinician, possibly the supervisor, make changes to their practice | 0.05 |
|  | 31 | It should address questions that are useful at a local level. Undergraduate research is unlikely to answer big clinical questions. | 0.21 |
|  | 36 | It should help choose between care pathways | 0.02 |
|  | 40 | It needs to be patient focused | 0.12 |
|  | 42 | The project itself must be on a clinically relevant topic | 0.15 |
|  | 53 | Any topic that looks at ways to improve the patient experience whether it be through social, psychological, or medical interventions. | 0.12 |
|  | 55 | Produce data that provides answers and/or opens up questions to help understand and evolve current practice. | 0.1 |
|  | 57 | links to current global health issues. | 0.27 |
|  | 62 | improve a doctor's clinical decision making or clinical skills. | 0.38 |
|  | 70 | A research project that would involve trialing different devices used in clinical medicine, such as ultrasound probes or different techniques | 0.36 |
|  | 71 | It should be useful - whether that be to other students, to clinicians, to other researchers. | 0.28 |
|  | 73 | Of current relevance relating to global/current events. For example COVID related research. | 0.28 |
|  | 77 | Be targeted to optimise the standard of patient care | 0.04 |
|  | 81 | Application to clinical diagnosis or treatment. It would ideally result in either the introduction of new diagnostic information or a change in standard of care | 0.03 |
|  | 83 | relate to an existing clinical need. | 0.05 |
|  | 88 | Research finding may potentially change the way in which patient care is received or the treatment options used. Therefore have a clinically significant addition of knowledge. | 0.02 |
|  | 89 | Shine light on Tx options | 0.04 |
|  | 91 | Answer a clear clinical need | 0.03 |
|  | 92 | Enhance understanding of clinical practice | 0.16 |
|  | 94 | enhance the clinical skillset/knowledge of other clinicians | 0.28 |
|  | 96 | Pragmatic and add to the knowledge base clinical care | 0.18 |

| **Cluster solution** | **STATEMENTS** | | **BRIDGING** |
| --- | --- | --- | --- |
| **Asking a Clinical Question** | | | 0.44 |
|  | 24 | Address a real world problem/issue | 0.25 |
|  | 32 | There should be emphasis on applied research outcomes | 0.52 |
|  | 41 | It has to address gaps in the literature | 0.67 |
|  | 56 | The results should provide information regarding the effectiveness of a distinct aspect or aspects of clinical practice identified prior to starting the project. | 0.25 |
|  | 61 | The research must analyse the benefits and the flaws of current practices and highlight exact areas of improvement in certain procedures specific to said research to allow for changes or implementations of new or revised regulations | 0.29 |
|  | 64 | Answer a specific question/need | 0.63 |
|  | 69 | There should be a very clear research question so that information that is collected can provide suggestions for further research or can guide or suggest small changes in practice that will benefit patients, healthcare professionals or both | 0.59 |
|  | 78 | Be sanctioned by a practicing clinical who identifies the need for it to be carried out | 0.55 |
|  | 87 | have outcomes which identify possible improvements to clinical care, or re-enforce existing practice. | 0.19 |
